# Osthole improves function of periodontitis periodontal ligament stem cells via epigenetic modification in cell sheets engineering

**DOI:** 10.1038/s41598-017-05762-7

**Published:** 2017-07-12

**Authors:** Jin Sun, Zhiwei Dong, Yang Zhang, Xiaoning He, Dongdong Fei, Fang Jin, Lin Yuan, Bei Li, Yan Jin

**Affiliations:** 1 State Key Laboratory of Military Stomatology & National Clinical Research Center for Oral Diseases & Shaanxi Key Laboratory of Oral Diseases, Center for Tissue Engineering, Fourth Military Medical University, Xi’an, Shaanxi 710032 China; 2 Xi’an Institute of Tissue Engineering and Regenerative Medicine, Xi’an, Shaanxi 710032 China; 3 Department of Stomatology, The Affiliated Shenzhen Maternity and Child Healthcare Hospital of the South Medical University, Shenzhen, Guangdong 518048 China; 4 Department of Oral and Maxillofacial surgery, General Hospital of Shenyang Military Area Command, Shenyang, Liaoning 110840 China; 5 Department of Orthopaedics, Xijing Hospital, the Fourth Military Medical University, Xi’an, Shaanxi 710032 China; 6 Department of Orthodontics, School of Stomatology, Fourth Military Medical University, Xi’an, Shaanxi 710032 China; 7 Department of Stomatology, the First Affiliated Hospital of Guangzhou Medical University, Guangzhou, Guangdong 510140 China

**Keywords:** Stem-cell differentiation, Molecular medicine

## Abstract

Inflammatory microenvironment causes the change of epigenetic modification in periodontal ligament stem cells derived from periodontitis tissues (P-PDLSCs), which results in defective osteogenic differentiation compared to cells from healthy tissues. It’s urgent to explore therapeutic strategies aimed at epigenetic targets associated with the regenerative ability of PDLSCs. Osthole, a small-molecule compound extracted from Chinese herbs, has been documented to promote osteogenesis and cell sheets formation of healthy PDLSCs. However, whether osthole shows same effect on P-PDLSCs and the mechanism of promotive effect is still unknown. The purpose of this study was to determine whether Osthole could restore defective osteogenic differentiation of P-PDLSCs via epigenetic modification. We demonstrated that 10^−7^ Mol/L of Osthole was the best concentration for osteogenic differentiation and proliferation of P-PDLSCs. Mechanistically, we also found that Osthole upregulated MOZ and MORF, histone acetylases that specifically catalyze acetylation of Histone3 lisine9 (H3K9) and Histone3 lisine14 (H3K14), which are key regulators in osteogenic differentiation of P-PDLSCs. Furthermore, Osthole treatment improved cell sheet formation and enhanced the bone formation of PDLSC sheets in animal models of periodontitis. Our study suggests that Osthole is a promising drug to cure periodontitis via regulating epigenetic modification in cell sheets engineering.

## Introduction

Periodontitis is an inflammatory disease that brings pathological alterations to the tooth-supporting tissues, consisting of gingiva, alveolar bone, periodontal ligament (PDL) and root cementum^1^, and the absorption occurring in alveolar bone is the biggest issue to be focused. It can affect up to 90% of the worldwide population. Periodontal ligament stem cells (PDLSCs) are a group of MSCs derived from periodontal ligament and PDLSCs were used for treating periodontitis in numerous preclinical and clinical studies^2^. However, defective osteogenic differentiation of PDLSCs were documented being closely related to periodontitis^3, 4^. Besides, osteogenic deficit of PDLSCs from periodontitis patients can not be recovered from *ex vivo* culture or expansion. Such deficiency of stem cells seems to retain a “memory” of abnormal microenvironment, which suggests the epigenetic modification may be involved. Recently, growing evidences also show the involvement of epigenetic modifications in the development of periodontal disease^5, 6^. It’s urgent to explore therapeutic strategies aimed at epigenetic targets associated with the regenerative ability of PDLSCs and the development of periodontitis.

Variations of epigenetic components result in the changes of specific chromatin architecture, then activate or deactivate expression of the regulatory genes, and finally impose influence on cell fate determination without changes of DNA sequences. Compared to genetic mutation, epigenetic modification is reversible and probable to be regulated by rules^7^. Growing evidences suggest that posttranscriptional modification of histone by acetylation plays an important role in osteogenic differentiation of PDLSCs^8^. Our previous study also showed that histone acetyltransferase GCN5 regulated the osteogenesis of PDLSCs and could be used as a target to treat periodontitis^9^. In addition, application of drugs that target epigenetic regulators in MSCs is being investigated^10, 11^. It has been proved that drugs restrained overwhelmingly inflammation by mean of modulating epigenetics^12^. Osthole, a small-molecule compound extracted from Chinese herbs, is a natural coumarin mainly contained in Cnidium monnieri. Osthole exhibits anti-oxidant and immunomodulatory properties, and is commonly applied in clinical practice of Traditional Chinese Medicine for cancer, inflammation etc.^13^. Previous study showed that owing to estrogen-like effects of Osthole, it prevents osteoporosis and reduces bone loss in ovariectomized rats by activation of β-catenin-BMP signaling^14^. Moreover, Osthole has been identified as one of the chromatin regulators implicated in the inhibition of histone deacetylases (HDACs) in order to cure or prevent cancer^15, 16^.

Cell sheet technology has been established as a promising concept for cell delivery that allows for a sheet of interconnected cells and cells in full contact with their natural extracellular environment to be delivered^17^. Periodontal ligament cell sheets are considered as a TE-biological complex which mimics the growing environment of periodontal ligament and facilitates cellular signal communications that could improve the regeneration of periodontal tissue. Our previous study showed that Osthole could promote extracellular matrix (ECM) deposition and osteogenic differentiation in PDLSC sheets^18^. However, whether osthole shows same effect on PDLSCs derived from periodontitis and the mechanism of promotive effect of Osthole are still unknown. Here, our study focused on investigating of the functional role and the molecular mechanism of Osthole, especially its regulation of epigenetic modification in reversing osteogenic defect of P-PDLSCs, and improving the regenerative ability of P-PDLSC sheets.

## Results

### Osteogenic effect of TCMs on P-PDLSCs

Previous studies showed that CatheRine Genistein, Xanthohumol, Osthole and Kaempferol are common single agents extracted from TCM and are able to be a cure for bone related diseases^22–24^. We isolated H-PDLSCs and P-PDLSCs as characterized by flow cytometry (Fig. S1). We applied these drugs to P-PDLSCs to determine the osteogenic induction ability. With two common used concentrations (10^−5^ and 10^−6^ Mol/L), ALP staining was performed to test whether agents mentioned above play a role in osteogenic differentiation of P-PDLSCs. Results in Fig. S2 showed that only Osthole significantly promoted ALP expression in P-PDLSCs while Xanthohumol, Kaempferol and CatheRine Genistein appeared to show no effect on the osteogenesis of P-PDLSCs.

### Osthole reverses defective osteogenic ability of P-PDLSCs

**Figure 1:**
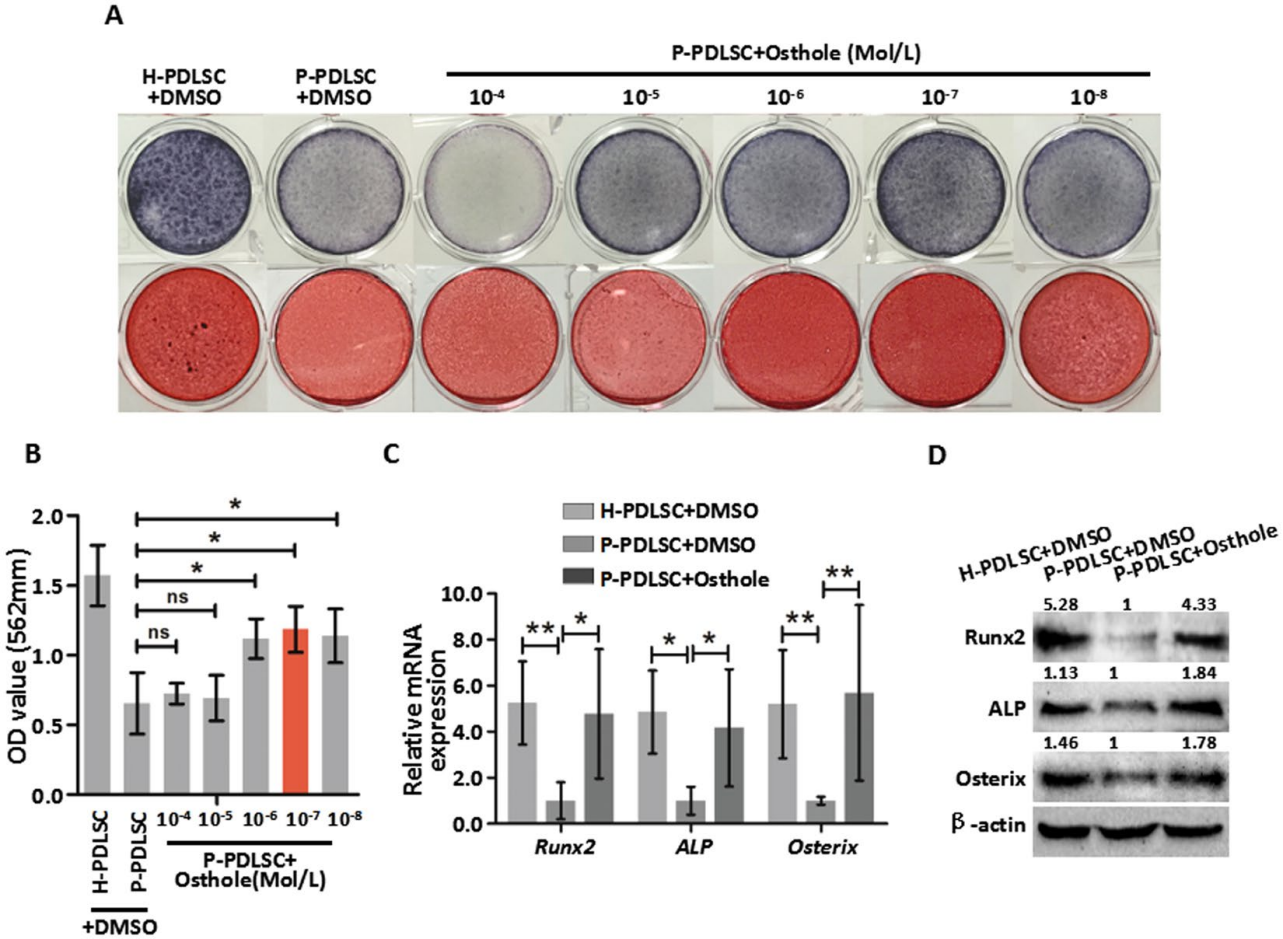
Osthole reverses defective osteogenic ability of P-PDLSCs. (**A**) ALP staining and ARS staining after 7 days (ALP staining) and 21 days (ARS staining) in H-PDLSCs and P-PDLSCs with different concentrations of Osthole (0 Mol/L, 10^−4^ Mol/L, 10^−5^ Mol/L, 10^−6^ Mol/L, 10^−7^ Mol/L and 10^−8^ Mol/L). (**B**) Quantification of ARS staining for light absorbance at 562 nm. (**C**) Gene expression of Runx2, ALP and Osterix in H-PDLSCs, P-PDLSCs and P-PDLSCs with 10^−7^ Mol/L Osthole as assayed by qRT-PCR. (**D**) Protein expression of Runx2, ALP and Osterix in H-PDLSCs, P-PDLSCs and P-PDLSCs with 10^−7^ Mol/L Osthole as assayed by Western blot. *P < 0.05, **P < 0.01, ns: *P* ≧ 0.05, n = 3.

To further investigate the role of Osthole on osteogenic differentiation of P-PDLSCs, different concentrations (10^−4^ to 10^−8^ Mol/L) of Osthole were applied to P-PDLSCs. 10^−4^ and 10^−5^ Mol/L Osthole suppressed proliferation of PDLSCs as shown by 3-(4,5-dimethylthiazol-2-yl)-2,5-diphe-nyltetrazolium bromide (MTT) assay after 3 days culture (Fig. S3). Meanwhile, ALP staining with series concentrations indicated that 10^−6^, 10^−7^and 10^−8^ Mol/L of Osthole appeared to increase ALP staining compared to DMSO group (Fig. 1A). Alizarin Red Staining showed that 10^−6^, 10^−7^and 10^−8^ Mol/L of Osthole increased the formation of mineralized nodules of P-PDLSCs (Fig. 1A,B). Noticeably, 10^−7^ Mol/L of Osthole was the most appropriate concentration for cell proliferation and osteogenic differentiation (Fig. 1A,B). In order to confirm whether 10^−7^ Mol/L of Osthole has an impact on the expression of osteogenic genes or proteins, we compared the expression of osteogenic genes or proteins between H-PDLSCs with DMSO, P-PDLSCs with DMSO and P-PDLSCs with 10^−7^ Mol/L of Osthole after osteogenic induction. Total RNA or protein were extracted from P-PDLSCs cultured for 14 days osteogenic condition with or without Osthole, and qRT-PCR or western blot analyses were performed to measure the expression of Runt-related transcription factor 2 (Runx2), ALP and Osterix. The results showed that 10^−7^ Mol/L of Osthole resulted in an obvious increase of the mRNA and proteins expression of Runx2, ALP and Osterix compared to the control group. The expression of osteogenic related genes and proteins in P-PDLSCs after Osthole treatment was similar to H-PDLSCs group (Fig. 1C,D). Synthetically, 10^−7^ Mol/L of Osthole were able to repair osteogenic deficiency in P-PDLSCs and was selected for the following tests.

### Osthole reverses defective osteogenesis of P-PDLSCs through histone acetylation

**Figure 2:**
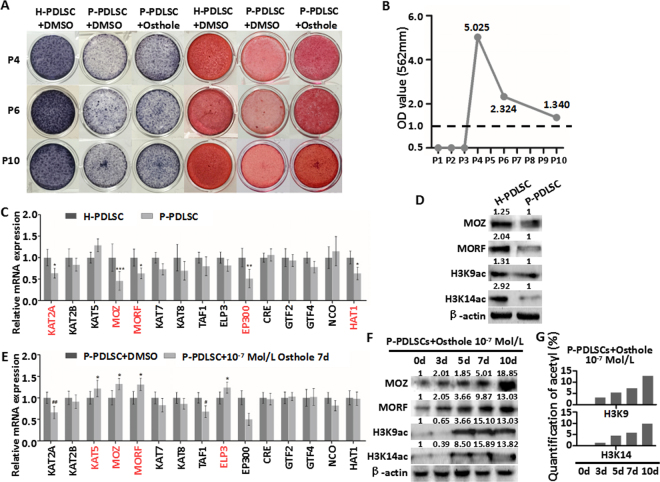
Osthole reverses defective osteogenesis of P-PDLSCs through histone acetylation. (**A**) ALP staining and ARS staining of H-PDLSCs, P-PDLSCs and P-PDLSCs with 10^−7^ Mol/L Ostholes in P4 (with stimulation), P6 (without stimulation) and P10 (without stimulation). (**B**) Quantification of ARS staining for light absorbance at 562 nm. (**C**) qRT-PCR showed gene expression of fifteen histone acetylases in H-PDLSCs and P-PDLSCs. (**D**) Protein expression of MOZ, MORF, H3K9ac and H3K14ac in H-PDLSCs and P-PDLSCs as assayed by western blot. (**E**) qRT-PCR showed gene expression of fifteen histone acetylases in P-PDLSCs and P-PDLSCs with 10^−7^ Mol/L Osthole measured by qRT-PCR. (**F**) Protein expression of MOZ, MORF, H3K9ac and H3K14ac in P-PDLSCs with 10^−7^ Mol/L Osthole treatment on day 0, 3, 5, 7, 10 as assayed by western blot. (**G**) Level of acetylation of H3K9 and H3K14 in P-PDLSCs with 10^−7^ Mol/L Osthole treatment on day 0, 3, 5, 7, 10 as assayed by EpiQuik Global Acetyl Histone Quantification Kit. *P < 0.05, **P < 0.01, ***P < 0.001, no mark: *P* ≧ 0.05, n = 3.

In order to know whether effect of Osthole on osteogenesis would last for consecutive passages, Osthole was applied to stimulate P-PDLSCs for 14 d before cells were passaged to next generation. Then, osteogenic differentiation was determined by ALP staining and Alizarin Red S staining after osteogenic induction for 21 days. The results revealed decreased osteogenic differentiation of P-PDLSCs compared to PDLSCs derived from healthy tissues after several passages (Fig. 2A,B). Meanwhile, the results also showed a sustained rescue effect of Osthole on osteogenic differentiation of P-PDLSCs after 14 days stimulation of 10^−7^ Mol/L of Osthole (Fig. 2A,B). These results indicated that Osthole might reverse defective osteogenesis of P-PDLSCs through epigenetic modification.

Since histone acetylation contributes to osteogenic differentiation of MSCs^19^, we profiled expression of 15 histone acetylases in H-PDLSCs and P-PDLSCs. The results showed that in comparison to H-PDLSCs, KAT2A, MOZ, MORF, EP300 and HAT1 were suppressed in P-PDLSCs as assayed by qRT-PCR analysis (Fig. 2C). The same histone acetylases were also profiled in P-PDLSCs with 10^−7^ Mol/L Osthole stimulation for 7 days. The results indicated that the expression of KAT5, MOZ, MORF and ELP3 were increased by Osthole treatment (Fig. 2E). After overlapping series of data from two tests above, the outcome revealed that both MOZ and MORF might play important role in rescuing the osteogenic differentiation of P-PDLSCs by Osthole treatment.

To further confirm the relationship between Osthole and MOZ/MORF, we detected the expression of MOZ/MORF and their major targets, H3K9ac and H3K14ac^20^. Western Blot showed that the expression of MOZ and MORF were decreased, while H3K9ac and H3K14ac were also decreased in P-PDLSCs compared to H-PDLSCs (Fig. 2D). MOZ and MORF, as well as H3K9ac and H3K14ac, were increased after Osthole treatment in P-PDLSCs (Fig. 2F). The results also showed that Osthole raised MOZ, MORF and their downstream targets in a time-dependent manner (Fig. 2F). Furthermore, EpiQuik Global Acetyl Histone Quantification Kit also indicated that level of acetylation of H3K9 and H3K14 increased along with time points after Osthole treatment (Fig. 2G). To summarize, MOZ and MORF might be related to osteogenic differentiation in PDLSCs via acetylation of H3K9 and H3K14, and Osthole might reverse defective osteogenesis of P-PDLSCs through MOZ and MORF.

### Decreased osteogenic differentiation of H-PDLSCs with MOZ & MORF depletion

**Figure 3:**
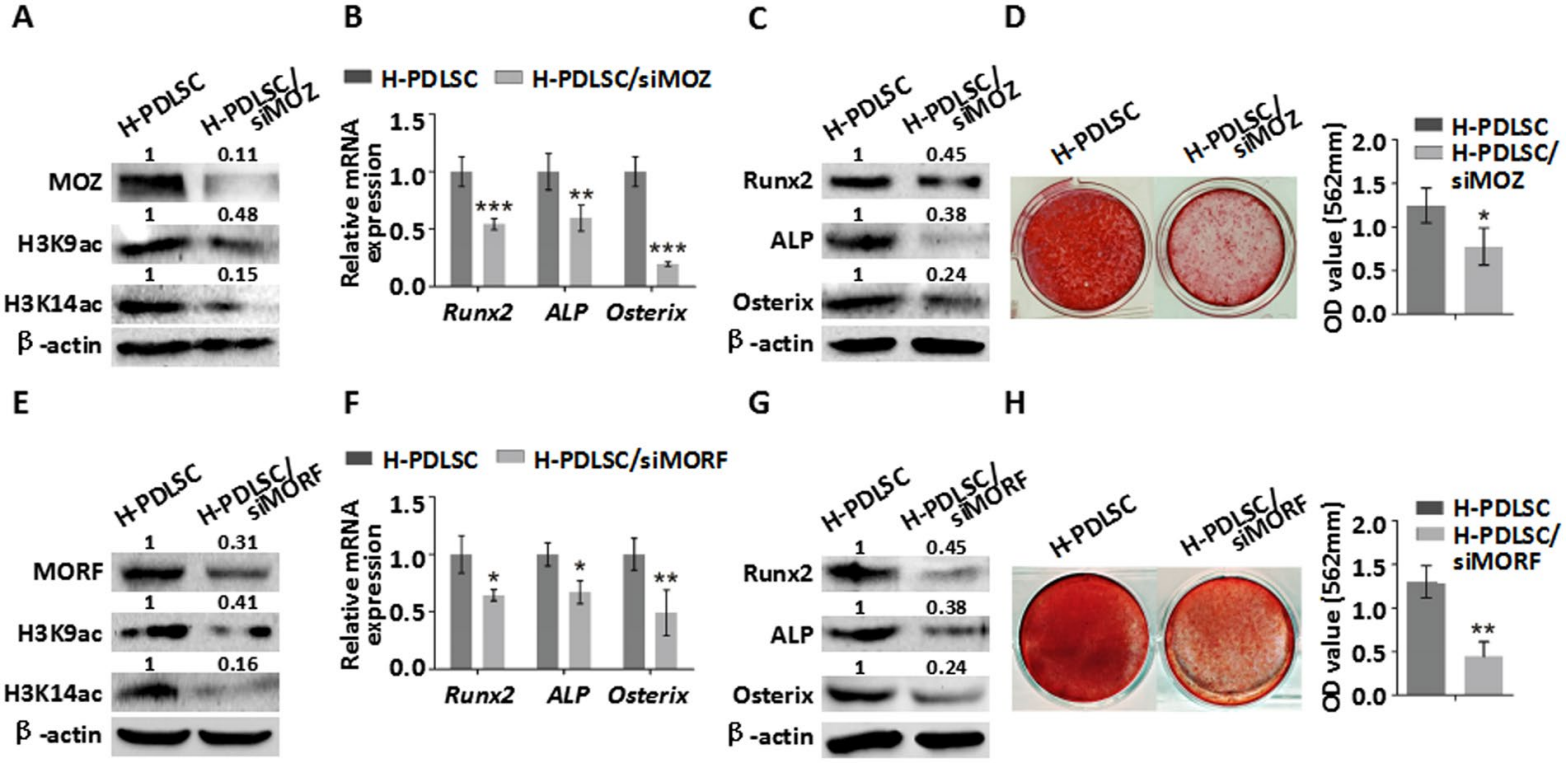
Osteogenic differentiation of H-PDLSCs with MOZ & MORF depletion. (**A**) Protein expression of MOZ, H3K9ac and H3K14ac in H-PDLSCs and H-PDLSCs with siMOZ. (**B**) Gene expression of Runx2, ALP and Osterix in H-PDLSCs and H-PDLSCs with siMOZ after osteogenic induction. (**C**) Protein expression of Runx2, ALP and Osterix in H-PDLSCs and H-PDLSCs with siMOZ after osteogenic induction. (**D**) ARS staining of H-PDLSCs and H-PDLSCs with siMOZ and quantification of it for light absorbance at 562 nm. (**E**) Protein expression of MORF, H3K9ac and H3K14ac in H-PDLSCs and H-PDLSCs with siMORF measured by western blot. (**F**) Gene expression of Runx2, ALP and Osterix in H-PDLSCs and H-PDLSCs with siMORF after osteogenic induction. (**G**) Protein expression of Runx2, ALP and Osterix in H-PDLSCs and H-PDLSCs with siMORF after osteogenic induction. (**H**) ARS staining of H-PDLSCs and H-PDLSCs with siMORF and quantification of it for light absorbance at 562 nm. *P < 0.05, **P < 0.01, ***P < 0.001, n = 3.

To further address whether MOZ and MORF influence the osteogenic differentiation of PDLSCs, we next examined the osteogenic differentiation of H-PDLSCs knocked down by small interfering RNA of MOZ or MORF (siMOZ or siMORF) separately. Western blot showed that siRNA of MOZ decreased the expression of MOZ, H3K9ac and H3K14ac in H-PDLSCs (Fig. 3A). The results also showed that siRNAs of MORF decreased the expression of MOZ, H3K9ac and H3K14ac in H-PDLSCs (Fig. 3E). Furthermore, we found that MOZ or MORF knockdown in H-PDLSCs reduced the expression of osteogenic differentiation related genes and proteins of Runx2, ALP and Osterix after 14 days osteogenic induction (Fig. 3B,C,F,G). Formation of mineralized nodules were also reduced after prolonged treatment with inducing media for 21 days after knockdown MOZ or MORF in H-PDLSCs (Fig. 3). These results showed that downregulated MOZ or MORF expression resulted in defective osteogenic differentiation in PDLSCs.

Since we found Osthole was beneficial to osteogenic differentiation in PDLSCs, we want to confirm if Osthole induces osteogenic differentiation of PDLSCs through MOZ or MORF. siRNAs were used before 10^−5^ Mol/L Osthole stimulation in H-PDLSCs. The results in Fig. S4A revealed that ALP activity of siMOZ or siMORF group was not increased with Osthole stimulation after 7 days induction. In addition, formation of mineralized nodules, as showed by Alizarin Red S staining and quantification, was also not increased in H-PDLSCs of MOZ or MORF knockdown with or without Osthole compared with control group after 21 days induction (Fig. S4B,C). These results confirmed that Osthole promotes osteogenic differentiation by means of epigenetic modification, especially through upregulation of MOZ or MORF.

### Osthole improves the formation and osteogenesis of P-PDLSC sheet

**Figure 4:**
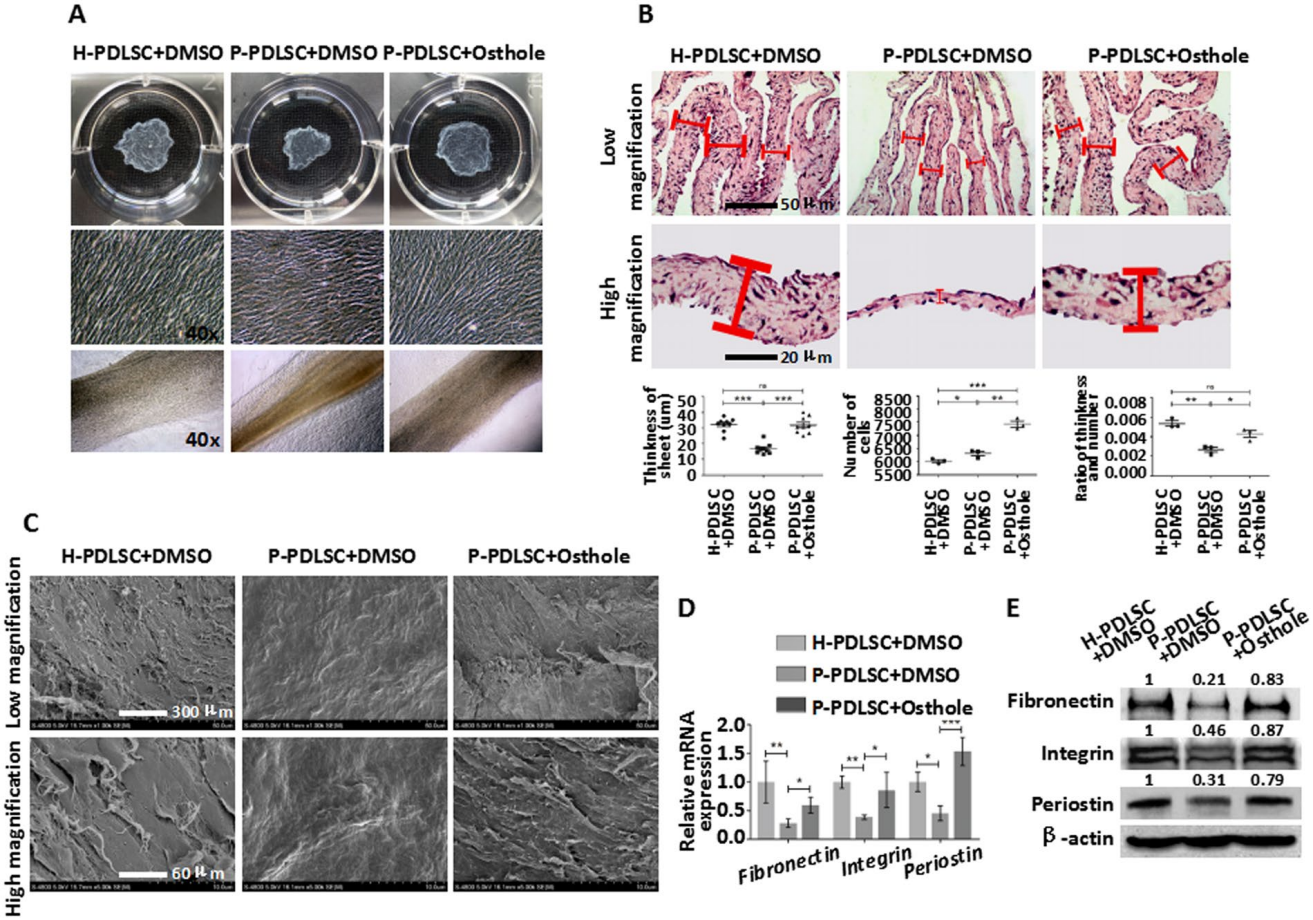
Osthole improves the formation of P-PDLSC sheet. (**A**) Representative macroscopic and microscopic images of H-PDLSC sheet, P-PDLSC sheet and P-PDLSC sheet with 10^−7^ Mol/L Osthole. (**B**) Representative H & E staining of H-PDLSC sheet, P-PDLSC sheet and P-PDLSC sheet with 10^−7^ Mol/L Osthole, and mean thickness of statistical analysis was shown below. Scale Bar, 50 μm (upper) and 20 μm (lower). (**C**) Representative SEM images of H-PDLSC sheet, P-PDLSC sheet and P-PDLSC sheet with 10^−7^ Mol/L Osthole. Scale Bar, 300 μm (upper) and 60 μm (lower). (**D**) Gene expression of Fibronectin, Integrin and Periostin in H-PDLSC sheet, P-PDLSC sheet and P-PDLSC sheet with 10^−7^ Mol/L Osthole as assayed by qRT-PCR. (**E**) Protein expression of Fibronectin, Integrin and Periostin in H-PDLSC sheet, P-PDLSC sheet and P-PDLSC sheet with 10^−7^ Mol/L Osthole as assayed by western blot. *P < 0.05, **P < 0.01, ***P < 0.001, ns: *P* ≧ 0.05, n = 3.

Our previous studies have revealed that Osthole could promote sheet formation of H-PDLSCs^18^. We investigated the promotional effect of Osthole on sheet formation of P-PDLSCs. After 10 days of culture with DMSO or Osthole all along, PDLSCs formed cell sheets that could be detached from the dishes. In view of forming of cell sheets, P-PDLSC sheet was smaller and less thickness than H-PDLSC sheet, meanwhile cells in P-PDLSCs presented worse organization and plasticity compared to H-PDLSCs (Fig. 4A). However, P-PDLSC sheet was improved when 10^−7^ Mol/L of Osthole was applied throughout the formation of sheet (Fig. 4A). In order to measure the thickness of sheets, which is one of necessary indexes for evaluating sheets, we fixed and cut them for Haematoxylin and eosin (H&E) staining. Microscope and measuring data demonstrated that mean thickness of P-PDLSC sheet with 10^−7^ Mol/L of Osthole exhibited much higher degree in thickness than P-PDLSC sheet with DMSO treatment no matter with or without exclusion of cells number (Fig. 4B). Furthermore, we utilized scanning electron microscope (SEM) to observe the micro-structure of sheets after 10 days culture of sheets. The outcome showed that P-PDLSC sheets treated with 10^−7^ Mol/L of Osthole established a higher density network and tightness junctions between cells from low-magnification microscope, and secreted more ECM from high-magnification microscope compared to P-PDLSC sheet with DMSO (Fig. 4C). In addition, both in genes and proteins level, Fibronectin, Integrin and Periostin, three representatives of ECM, were upregulated when sheet stimulated by 10^−7^ Mol/L of Osthole (Fig. 4D,E). Collectively, these data indicated that Osthole could reverse the defective ability of cell sheet formation in P-PDLSCs, and the effect after improvement was similar to PDLSCs derived from healthy tissues.

**Figure 5:**
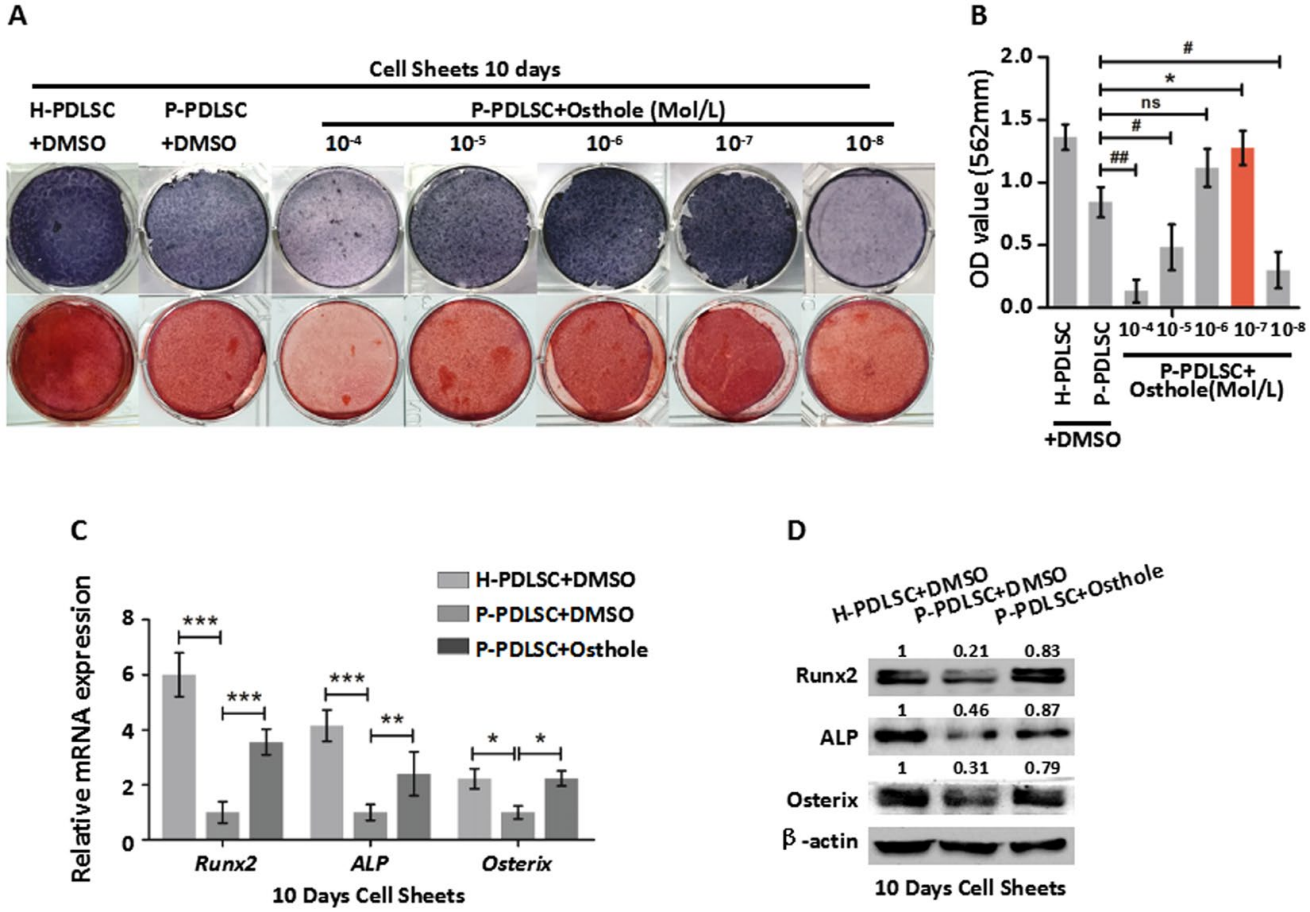
Osthole improves osteogenesis of P-PDLSC sheet. (**A**) ALP staining and ARS staining after 7 days (ALP staining) and 21 days (ARS staining) in H-PDLSC and P-PDLSC sheets with different concentrations of Osthole. (**B**) Quantification of ARS staining for light absorbance at 562 nm. (**C**) Gene expression of Runx2, ALP and Osterix in H-PDLSC sheet, P-PDLSC sheet and P-PDLSC sheet with 10^−7^ Mol/L Osthole. (**D**) Protein expression of Runx2, ALP and Osterix in H-PDLSC sheet, P-PDLSC sheet and P-PDLSC sheet with 10^−7^ Mol/L Osthole. */#*P* < 0.05, **/##*P* < 0.01, ***P < 0.001, ns: *P* ≧ 0.05, n = 3.

In order to examine whether Osthole improve osteogenic differentiation in P-PDLSC sheet, we constructed H-PDLSCs sheet and treated the sheet with different concentration of Osthole. Results declared that after 7 days osteogenic induction following 10 days culture with Vitamin C, P-PDLSCs in 10^−4^ Mol/L group had lower ALP expression to a certain degree while 10^−5^, 10^−6^ and 10^−7^ Mol/L of Osthole represented increased ALP expression (Fig. 5A). Alizarin Red Staining indicated a similar tendency in day 14 after osteogenic induction when sheets has formed (Fig. 5A,B). qRT-PCR or western blot analysis were performed to measure the expression of Runx2, ALP and Osterix. The results showed 10^−7^ Mol/L of Osthole resulted in a marked increase of the mRNA and proteins expression of Runx2, ALP and Osterix compared with the control group (Fig. 5C,D). This observation revealed that Osthole improved osteogenesis of P-PDLSC sheet.

### Osthole upregulates the expression of MOZ and MORF in P-PDLSC sheet

**Figure 6:**
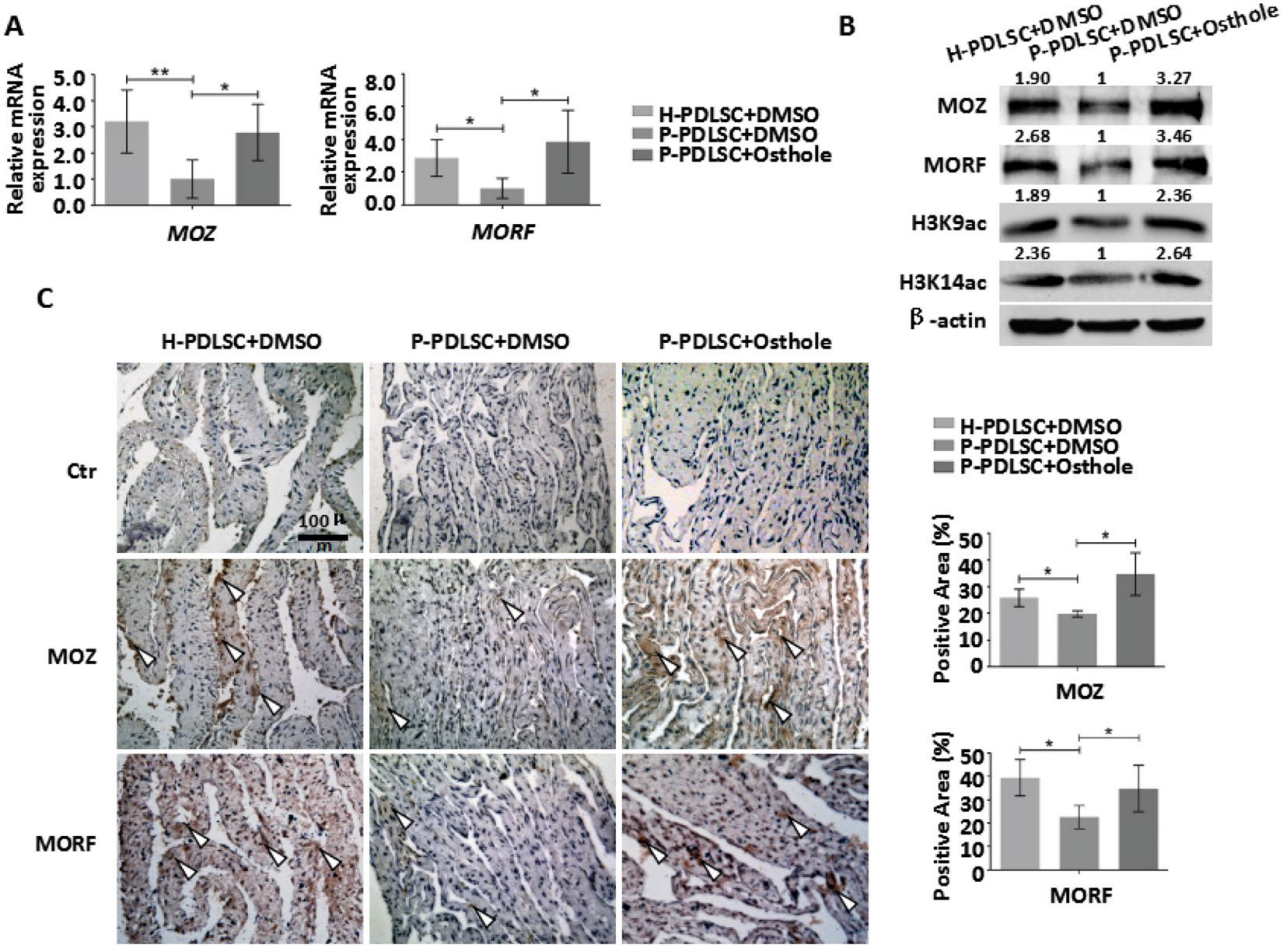
Expression of MOZ and MORF in PDLSC sheets with Osthole treatment. (**A**) Gene expression of MOZ and MORF in H-PDLSC sheet, P-PDLSC sheet and P-PDLSC sheet with 10^−7^ Mol/L Osthole. (**B**) Protein expression of MOZ, MORF, H3K9ac and H3K14ac in H-PDLSC sheet, P-PDLSC sheet and P-PDLSC sheet with 10^−7^ Mol/L Osthole. (**C**) Representative images of immunohistochemistry staining of MOZ and MORF in H-PDLSC sheet, P-PDLSC sheet and P-PDLSC sheet with 10^−7^ Mol/L Osthole. Semi-quantification of the positive area of MOZ and MORF was calculated. Scale Bar, 100 μm. *P < 0.05, **P < 0.01, n = 3.

Since previous results have proved that Osthole promotes osteogenic differentiation through upregulation of MOZ or MORF, we verified whether Osthole would exert effect on MOZ or MORF in P-PDLSC sheet. MOZ and MORF were detected after sheets formation with or without 10^−7^ Mol/L of Osthole stimulation. qRT-PCR and Western blot both revealed that MOZ and MORF were promoted in P-PDLSCs + Osthole group, similar to H-PDLSCs + DMSO group (Fig. 6A,B). Meanwhile, H3K9ac and H3K14ac were also upregulated in P-PDLSCs + Osthole group, which were consistent with the expression of MOZ and MORF (Fig. 6B). Furthermore, immunohistochemistry results of the sheets also showed more MOZ and MORF positive cells in P-PDLSC sheet with Osthole treatment compared to P-PDLSC sheet with DMSO (Fig. 6C). The results showed that Osthole upregulated the expression of MOZ and MORF in P-PDLSC sheet.

### Osthole treated P-PDLSC sheet shows improved bone formation in animal model

**Figure 7:**
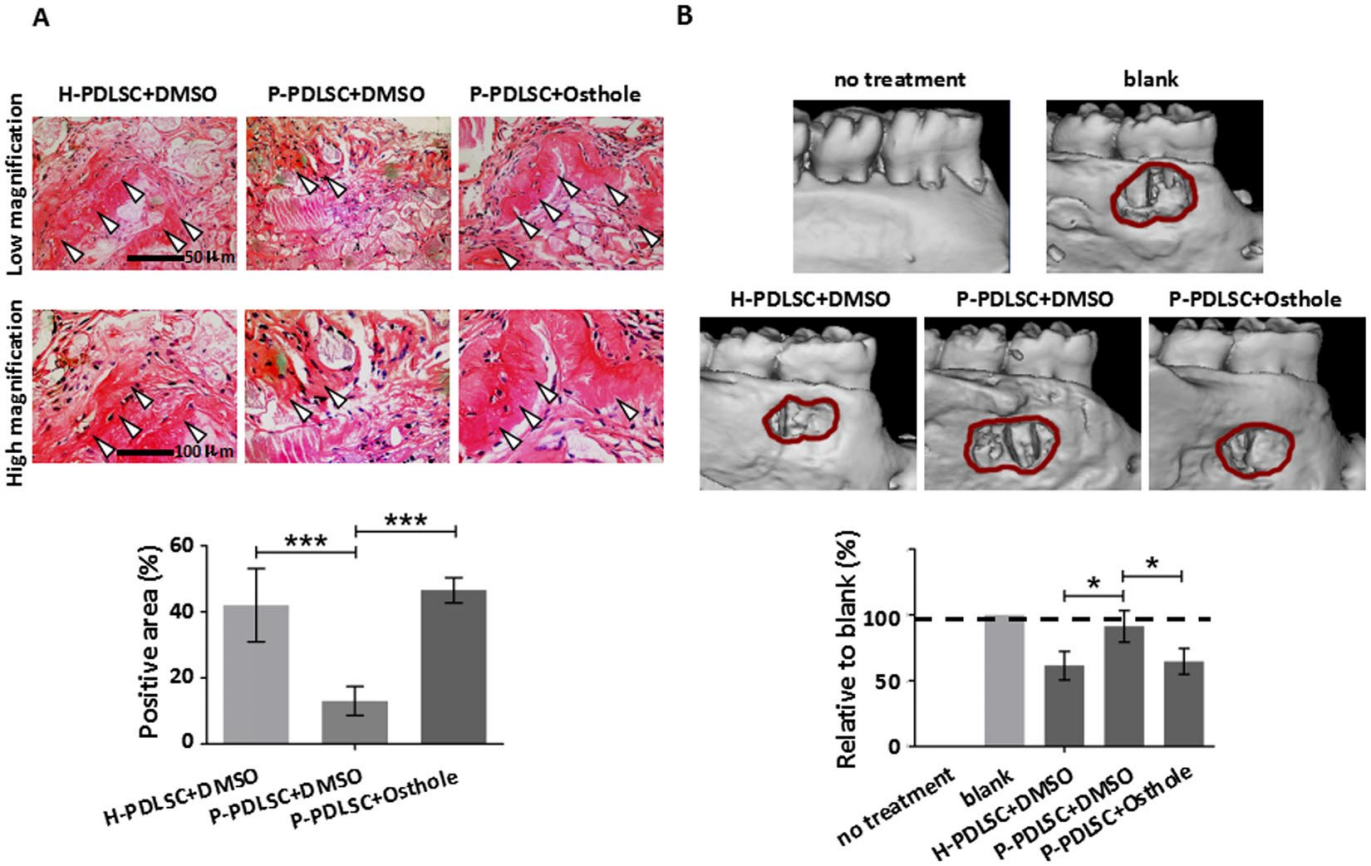
Osthole treated P-PDLSC sheet shows enhanced bone formation in animal model. (**A**) Representative images of H & E staining of H-PDLSC sheet, P-PDLSC sheet and P-PDLSC sheet with 10^−7^ Mol/L Osthole transplants in nude mice (arrow: new bone). Quantification of regenerated bone was calculated. Scale Bar, 50 μm (upper) and 100 μm (lower). (**B**) Micro CT analysis showed repair of bone defect in SD rats after transplantation of H-PDLSC sheet, P-PDLSC sheet and P-PDLSC sheet with 10^−7^ Mol/L Osthole. *P < 0.05, ***P < 0.001, n = 3.

In order to confirm the promotive osteogenesis of Osthole on P-PDLSC sheet, we carried out *in vivo* experiments with animal model. From the results of H & E staining after different sheets with CBB transplanted in nude mice, H-PDLSCs + DMSO group displayed the strongest ability of osteogenesis in accordance with new bone formation (Fig. 7A). P-PDLSCs + DEMO group has the least formation of new bone compared with others, while P-PDLSCs added 10^−7^ Mol/L of Osthole showed a distinct increase of new bone formation (Fig. 7A), which was similar to H-PDLSCs with DMSO.

Then, in the inferior alveolar defect mode by 3D reconstruction, we observed that P-PDLSCs sheet after 10^−7^ Mol/L of Osthole treatment exhibited obviously enhanced restoration of the bone defect (Fig. 7B). And P-PDLSCs + DMSO showed no improvement of the defect (Fig. 7B), which suggested that osteogenic ability of P-PDLSC sheet with Osthole was enhanced *in vivo*, which could be used as a strategy to treat periodontitis.

## Discussion

In this study, we provided a strategy with epigenetic modification from Chinese herb to reverse osteogenic differentiation of cells and improve formation of cell sheet from periodontitis-derived PDLSCs. Till today, many Chinese herbs have been proved to be effective in treating osteoporosis and immuno-disorders since Chinese herbs could act as therapeutic agents and mediators of stem cell differentiation and rejuvenation^21^. There are researches reporting that Genistein, Xanthohumol, Osthole or Kaempferol modulate expression of osteoblast-specific genes or inhibit osteoclastic differentiation in adult stem cells or animals^22–24^. In light of recent researches, we found that only Osthole specially promoted osteogenic differentiation of P-PDLSCs compared to other small-molecule compounds extracted from Chinese herbs. It reminded us that Osthole could be a valuable promoter in bone tissue engineering field.

Osthole is a natural coumarin extracted from Cnidium monnieri or Angelica pubescens. Since its structural formula is highly similar to estrogen of mammals and it has pharmacological activities of immunoregulation, anti-inflammatory, anti-oxidant and anti-cancer effects^25^, Osthole has received considerable attention to be a promising compound for new drug discovery and therapeutic application. Our previous study found that 10^−5^ Mol/L of Osthole accelerated cell sheet formation and osteogenic genes/proteins expression in both PDLSCs and MSCs from jaw bone marrow (JBMMSCs)^18^, but its effect on PDLSCs from periodontitis patients and the molecular mechanism of its promotive effect on osteogenesis are remained largely unknown. In our study, we found that 10^−7^ Mol/L was the best concentration for osteogenic differentiation and proliferation of P-PDLSCs as 10^−7^ Mol/L of Osthole dramatically increased ALP activity, calcium deposition and osteogenic genes/proteins expression compared to other concentrations. Taken together, Osthole of appropriate concentration not only promotes osteogenic differentiation of MSCs from healthy tissues, but also enhances osteogenesis of MSCs from pathological tissues. This finding might be more instructive for treatment of bone related diseases.

Epigenetic modification leads to the activation or repression of genes without changes of DNA sequence^26^. Such alteration of genes expression induced by microenvironment can be inherited during cell passages, resulting in maintenance of the acquired phenotype^33^. From the results in Fig. 2, after passing cells to P4, P6 and P10, P-PDLSCs showed osteogenic defect compared to PDLSCs derived from healthy tissues. It revealed that pathological microenvironment has already caused epigenetic modification in PDLSCs. At the same time, these data also indicated that 10^−7^ Mol/L of Osthole could rescue osteogenic defect of P-PDLSCs and the rescue effect lasts within passages. Emerging evidence suggests that lysine acetylation facilitates post-translational modification, and histone acetylation contributes to or regulate osteogenic differentiation of MSCs^27^. Furthermore, we found out that MOZ, MORF, H3K9ac and H3K14ac were decreased in P-PDLSCs and they were recovered when 10^−7^ Mol/L of Osthole stimulation was present. Meanwhile, osteogenic differentiation was reduced when MOZ or MORF was knockdowned in H-PDLSCs with or without Osthole treatment. These findings indicate that MOZ and MORF play important role in osteogenic differentiation, and they are key factors between Osthole and osteogenic differentiation of PDLSCs. Previous studies have showed that MOZ and MORF are critical for bone development and osteogenic genes expression^28–30^. MOZ and MORF are also lysine-specific acetylases that specifically catalyze acetylation of H3K9 and H3K14^20^. Therefore it is reasonable to assume that upregulated MOZ and MORF may acetylate H3K9 and H3K14 which bind to promoter of osteogenic genes, hence promoting osteogenic differentiation in P-PDLSCs treated with Osthole.

Recent clinically available techniques, such as open flap debridement with guided tissue regeneration (GTR) technology combining bone substitutes, biological mediators, or specific growth factors, are able to promote restoration of bone defect to a certain degree^31^. However, the treatments above still remain an issue that lack of complete and functional construction of damaged bone tissues^32^. An advanced concept of tissue engineering and regenerative medicine coupled with stem cells and specific factors in order to enhance bone tissues reconstruction is increasingly proved its promising prospect in both the clinical and research^2^. Although current applications with tissue engineering have already obtained conspicuous achievement, it would be profounder to seek out effective factors and determine their mechanisms. In our study, we demonstrate that 10^−7^ Mol/L of Osthole processes biological effects of promoting osteogenesis and extracellular matrices (ECM) production of P-PDLSCs sheet, which shows optimized characteristic similar to cells sheet derived from healthy tissues. 10^−7^ Mol/L of Osthole markedly enhances ALP activity, calcium deposition and osteogenic genes/proteins expression of PDLSCs. Meanwhile, it is able to accelerate cell sheet formation, which possesses high cell quantity, thickness and ECM production. Furthermore, previous research has revealed that cell sheet mineralization is in company of ECM production^18^. However, 10^−8^ osthole treatment, which increased the osteogenesis of PDLSCs, did not show increased calcium deposition of PDLSCs sheet. This result indicates that cells and cell sheets show different responses when receive 10^−8^ osthole treatment. Taken together, it is logical to believe that Osthole at an appropriate concentration advances cells sheet technology of restoring bone defect via stimulating cell sheet to improve new bone and bone-like matrix regeneration.

To confirm the relationship between epigenetics and regeneration, we verified expression of MOZ and MORF after cell sheet formation treated with 10^−7^ Mol/L of Osthole. The results indicate that MOZ and MORF are upregulated in Osthole group at gene and protein level, and H3K9ac and H3K14ac are increased at protein level. Meanwhile, immunohistochemistry staining revealed that expression of MOZ and MORF were increased in cell sheet formation with Osthole stimulation. Altogether, improved osteogenesis is positive associated with MOZ and MORF in cell sheet. Moreover, Osthole treated P-PDLSCs sheets improve the ectopic bone regeneration in nude mice and enhance bone formation in the inferior alveolar defect of rats. These data indicate Osthole is applicable in improvement of cell sheets and stem cell-based periodontal regeneration.

To summarize, we demonstrate that Osthole promotes osteogenic differentiaiton of P-PDLSCs and ECM production of P-PDLSCs sheet *in vitro* and *vivo*. Moreover, we find that Osthole regulates the expression of MOZ and MORF which are critical in osteogenic differentiaiton of P-PDLSCs. Interestingly, we reveal that Osthole possesses enduring effect existing in P-PDLSCs because of epigenetic modification. These results provide a strategy of Osthole at first time for stem cell sheet engineering and regenerative medicine via epigenetic modification.

## Materials and Methods

### Cell culture

Healthy human teeth samples were collected from individuals (n = 5, aged from 16 to 30 years) extracted for orthodontic reason. The standard of healthy periodontal tissues: no bleeding on probing, probing depth ≤4 mm, alveolar bone loss ≤3 mm. Teeth affected by periodontitis were collected from periodontitis patients, (n = 5, aged 30–45 years). The clinical diagnosis of chronic periodontitis: more than 1 pocket probing depth >5 mm, alveolar bone loss >2/3 length of teeth. The subjects engaged in this study had no history of systemic diseases, smoking or taking special medicine over the past half year. Written informed consent were provided by all participants, and ethical approval had been obtained from the Ethics Committee of the School of Stomatology, the Fourth Military Medical University. All the methods in the study were carried out in accordance with the approved guidelines.

PDLSCs were isolated and cultured as we previously described^9^. Briefly, after separation the middle part of the root surface, the periodontal ligament (PDL) was enzymatically digested with type I collagenase (0.66 mg/ml; Sigma, St Louis, MO, USA) for 1 hr (suspended every 10 mins), and then cultured in α-MEM (Invitrogen, Carlsbad, CA, USA) supplemented with 10% fetal bovine serum (FBS; Thermo Electron, Melbourne, Australia), 0.292 mg/ml glutamine (Invitrogen, Carlsbad, CA, USA), 100 U/ml penicillin, and 100 mg/ml streptomycin in 6-well culture dishes (Costar, USA) at 37 °C in a humidified atmosphere of 5% CO_2_ and 95% air. After 2 weeks in culture, cells from PDL became subconfluent. To obtain homogeneous populations of PDLSCs, single-cell-derived colonies were obtained using the limiting dilution technique. Single-cell-derived colonies were used in passage 3–5. For each experiment, PDLSCs in the same passage were used.

### Flowcytometric characterization of surface markers expression pattern on PDLSCs

PDLSCs were stained with antibodies for stem cell surface markers and analyzed by flow cytometry. Briefly, to identify the phenotypes of PDLSCs, 5 × 10^5^ cells at the passage 3 were incubated with phycoerythrin (PE) conjugated monoclonal antibodies for human CD146, CD34 (Biolegend, San Diego, CA, USA), CD90, CD14 (eBioscience, San Diego, CA, USA) and fluorescein isothiocynante (FITC) conjugated monoclonal antibodies for STRO-1, CD105 (eBioscience, San Diego, CA, USA) as the manufacturer’s instructions. The incubation procedure was carried out at 4 °C away from light for 1 hour. After washing with PBS, cells were subjected to flow cytometric analysis (Beckman Coulter, Fullerton, CA, USA).

### Cell sheet culture

1 × 10^6^ cells per well were seeded in 6-well dishes for 24 hrs subsequently incubated with cell sheet-induction medium (50 μg/mL vitamin C) for cell sheet production. Meanwhile, containing DMSO or Osthole with different contributions were according to the group design. Induction medium with stimulation was changed every 3 days. After 14 days, the resulting cell sheets were taken photos by a CDD camera or a light microscope (BX-51, Olympus, Japan). Then cell sheets were harvested using sterile tweezers and fixed in 4% paraformaldehyde for 12 hrs for the following experiments, or stained with ARS (sheets were continued 14 days osteogenic induction) or ALP (sheets were continued 7 days osteogenic induction) directly, or extracted RNA or proteins for qRT-PCR or western blot.

### Small-molecule-compounds application

To characterize and compare the osteogenic effect of Genistein, Xanthohumol, Osthole and kaempferol (National Institutes for Food and Drug Control, Beijing, China) on P-PDLSCs, 1 × 10^5^ cells per well were maintained in 12-well dishes for 24 hrs subsequently incubated with 10^−5^ Mol/L, 10^−6^ Mol/L Genistein, Xanthohumol, Osthole or kaempferol or DMSO with equal quantity in α-MEM for 48 hrs^18^. Then culture conditions were replaced with osteogenic induction medium (100 nM dexamethasone, 50 mg/ml ascorbic acid, and 5 mM b-glycerophosphate; Sigma, St Louis, MO, USA) with 10^−5^, 10^−6^ Genistein, Xanthohumol, Osthole or kaempferol or DMSO with equal quantity all along for 7 days or 21 days for ALP staining or ARS staining. Osteogenic induction medium with small-molecule-compounds was refreshed every 3 days.

### Cellular toxin assay

To identify concentrations of Osthole that would positively influence P-PDLSC behavior, based on previous study^18^, we selected 6 concentrations (0 Mol/L, 10^−4^ Mol/L, 10^−5^ Mol/L, 10^−6^ Mol/L, 10^−7^ Mol/L and 10^−7^ Mol/L for the 3-(4,5-dimethylthiazol-2yl)-2,5-diphenyltetrazolium bromide (MTT) assay. Cells were maintained in 96-well culture dishes at a concentration of 2 × 10^3^ per well. After growing in α-MEM for 24 h, medium was replaced with α-MEM containing different concentrations of Osthole all along. Each concentration group included 5 wells. MTT assay was carried out each day of a 7-day culture period. Briefly, 20 mL of 5 mg/mL MTT solution (Sigma Aldrich, 5 mg/mL) was added to each well and incubated for 4 h. The medium was discarded, formazan salts were dissolved with vibration in 150 mL dimethylsulfoxide (DMSO) (Sigma Aldrich), and the dishes were read at 490 nm by a microplate reader (ELx800, BioTek Instruments Inc., Highland Park, USA). Statistical images were presented by Prism 5.0 (GraphPad Software, La Jolla, CA, USA).

### Alkaline phosphatase (ALP) staining

After osteogenic induction for 7 days, cells were rinsed two times with phosphate-buffered saline (PBS). Then nitro blue tetrazolium (NBT) and 5-bromo-4-chroro-3-indolyl phosphate (BCIP) (Beyotime, Shanghai, China) were added into cells for the staining. 30 min later, staining agent was discarded and cells were rinsed two times with PBS in order to preclude non-positive staining. Images of each sample were acquired using a CDD camera.

### Alizarin Red S (ARS) staining

After osteogenic induction for 21 days, cells were rinsed two times with PBS. Then 1% Alizarin Red S (Kermel, Tianjin, China) were added into cells for the staining. 1 min later, staining agent was discarded and cells were rinsed two times with PBS in order to except non-positive staining. Images of each sample were acquired using a CDD camera. To quantify matrix mineralization, Alizarin red S-stained cells were incubated in 100 mM cetylpyridinium chloride through vibration to solubilize and release calcium-bound Alizarin red S into the solution. The absorbance of the released Alizarin red S was measured at 562 nm. Relative Alizarin red S intensity was calculated according to the absorbance and presented by Prism 5.0.

To evaluate the effect of Osthole at a concentration of 10^−7^ Mol/L on the osteogenic differentiation of P-PDLSCs, we designed 3 testing groups, H-PDLSCs + DMSO, P-PDLSCs + DMSO and P-PDLSCs + Osthole (10^−7^ Mol/L). Briefly, cells (P3) were maintained into bottles and cultured until the cells reached 80% confluence, then subjected to DMSO or Osthole stimulation for 14 days. Stimulation medium was refreshed once every 3 days. After the stimulation, cells were passed to P4, P6 and P10. To examine osteogenic potential, cells of 3 groups in P4, P6 and P10 (each at a density of 1 × 10^5^ cells/well) were maintained into 12-well dishes separately and further induced with osteogenic medium for 7 days or 21 days in the same condition for ALP staining or ARS staining. According to the absorbance of the released Alizarin Red S, graph was made by ratio of P-PDLSCs + Osthole group and P-PDLSCs + DMSO group by Prism 5.0.

### Total RNA extraction and Quantitive Real-time reverse transcriptase-polymerase chain reaction (qRT-PCR)

Total cellular RNA was extracted by TRIzol reagent (Invitrogen, Carlsbad, CA, USA) according to the manufacturer’s instructions. Isolated total RNA was then subjected to reverse transcription using OligodT primer and PrimeScript® RTase (Takara, Dalian, China) according to the manufacturer’s instructions. qRT-PCR was performed with SYBR® Premix Ex Taq™ II (Takara, Dalian, China) using the C1000TM Thermal Cycler (Bio-Rad, Hercules, CA, USA). The expression levels of the target genes were normalized to that of the housekeeping gene GAPDH. Images were presented by Prism 5.0. The sequences of primers used are shown in Supplementary Table 1.

### Protein isolation and western blot analysis

Total proteins were extracted with lysis buffer (10 mM Tris–HCl, 1 mM ethylenediaminetetraacetic acid (EDTA), 1% sodium dodecyl sulfate, 1% Nonidet P-40, 1:100 proteinase inhibitor cocktail, 50 mM β-glycerophosphate, 50 mM sodium fluoride) (Beyotime, Shanghai, China). The protein concentration was determined with a protein assay kit (Beyotime) following the manufacturer’s instructions. Aliquots of 40 μg per sample were separated by 10% sodium dodecyl sulfate-polyacrylamide gel electrophoresis (SDS-PAGE), transferred to polyvinylidene fluoride (PVDF) membranes (Millipore, Billerica, MA, USA) and blocked with 5% bovine serum albumin (BSA) in PBST (PBS with 0.1% Tween), then incubated with the following primary antibodies overnight: anti-ALP (#ab65834), anti-Osterix (#ab187158), anti-H3K9Ac (#ab10812), anti-H3K14Ac (#ab52946), anti-Fibronectin (#ab32419), anti-Periostin (#ab92460) (Abcam, Cambridge, UK), anti-MOZ (#sc-5713), anti-MORF (#sc-368690) (Santa Cruz Biotechnology, CA, USA), anti-Runx2 (#12556), anti-Integrin (#9699) (Cell Signaling Technology, Beverly, MA, USA), anti-β-actin (#CW0100, CWBIO, China). Then, the membranes were incubated with horseradish peroxidase-conjugated secondary antibody (Boster, Wuhan, China). The blots were visualized using an enhanced chemiluminescence kit (Amersham Biosciences, Piscataway, NJ, USA) according to the manufacturer’s instructions. Western blot intensity was calculated by Image-Pro Plus 6.0 (media cybernetics, USA) according to the ratio of experimental group to control group corrected by β-actin.

### Small interfering RNA (siRNA) and transfection assays

For siRNA inhibition studies, PDLSCs were grown to 60% confluence. SiRNA against human MOZ, MORF and a negative control (Ribobio Co., China) were transfected into cells at a final concentration of 50 nM using the Lipo2000 (Invitrogen, Carlsbad, CA, USA) according to the manufacturer’s instructions. After transfection, the cells were harvested at 48 h for protein extraction or the medium was changed to osteogenic medium with Osthole for the following experiments.

### Acetylation of H3K9 and H3K14

Total proteins were extracted with lysis buffer (10 mM Tris–HCl, 1 mM ethylenediaminetetraacetic acid (EDTA), 1% sodium dodecyl sulfate, 1% Nonidet P-40, 1:100 proteinase inhibitor cocktail, 50 mM β-glycerophosphate, 50 mM sodium fluoride) (Beyotime, Shanghai, China). The acetylation of H3K9, and H3K14 was determined with EpiQuikTM Global Acetyl Histone H3K9 Quantification Kit and EpiQuikTM Global Acetyl Histone H3K14 Quantification Kit (Epigentek, Farmingdale, NY, USA) following the manufacturer’s instructions.

### Haematoxylin and eosin (H & E) staining

Harvested PDLSC sheets from 3 group were fixed with 4% paraformaldehyde at 4 °C overnight. Then, the samples were rinsed three times with PBS. The samples were sectioned along the vertical direction of the sheets every 5 μm and stained with H & E by H & E kit (Labest, China).

Specimens were observed under a light microscope, and images of each sample were acquired randomly using a CDD camera. Quantification of the thickness of cell sheets was carried out using the software of Image-Pro Plus 6.0 (Media Cybernetics, USA), and images were presented by Prism 5.0.

### Scanning electron microscope (SEM)

Cell sheets were fixed in 2.5% glutaraldehyde and dehydrated gradually in ethanol (from 30 to 100%). The samples were further dried under critical point conditions and sputter coated with platinum. Observations were then performed using a scanning electron microscope (SEM; S-4800; Hitachi, Ltd., Tokyo, Japan).

### Ectopic bone regeneration assays

The use of nude mice for research was approved by the IRB of FMMU. The surgical procedures were performed according to the guidelines of the Animal Care Committee of FMMU. Nine 8-week-old male nude mice were purchased from the FMMU Animal Center. The *in vivo* bone formation capacity of PDLSC sheets derived from different pre-incubated cells (H-PDLSCs + DMSO, P-PDLSCs + DMSO and P-PDLSCs + Osthole (10^−7^ Mol/L) was investigated based on an ectopic transplantation model^18^. The transplants were composed of 15 mg of calcined bovine bone (CBB) wrapped in 4-fold sheets.

1% pentobarbital sodium solution (0.1 mL/100 g) was used for intraperitoneal anesthesia of nude mice. The transplants were subcutaneously transplanted into the dorsal region of nine 8-week-old male nude mice with 3 nude mice in each group. After 2 months, the mice were euthanized and the excised tissue samples were fixed in 4% paraformaldehyde for 24 hrs and then the samples were decalcified with 10% ethylene diamine tetraacetic acid (EDTA) for 1 month. The obtained samples were subjected to H & E staining. The percentage of new bone area in the total area was calculated from 3 randomly selected fields from each specimen with the aid of Image-Pro Plus 6.0. The mean percentage of new bone area in the total area was obtained for the statistical analysis. Statistical images were presented by Prism 5.0.

### Inferior alveolar defect mode

The use of Sprague-Dawley (SD) male rats for research was approved by the IRB of FMMU. The surgical procedures were performed according to the guidelines of the Animal Care Committee of FMMU. Fifteen SD rats (weighing 180 ± 15 g) from the FMMU Animal Center were randomly divided into 5 groups, with 3 animals in each group. 1% pentobarbital sodium solution (0.1 mL/100 g) was used for intraperitoneal anesthesia of SD rats.The skin at the surgical site was shaved and disinfected with iodophor. An incision through the full thickness of the skin was made along the inferior border of the unilateral mandible and the masseter muscle and periosteum were removed to expose the buccal surface of the mandible. An inverted cone bur, supported with copious saline irrigation, was used to remove the alveolar bone and cementum covering the roots of the mandibular molars, resulting in a periodontal defect (approximately 3 mm × 2 mm). These defects were filled with sheet/CBB constructs (sheets obtained from 6-well culture dishes, folded 4 times, wrapping 3 mg of CBB). PDLSC sheets were derived from 3 different pre-incubated cells as described in the group design (H-PDLSCs + DMSO, P-PDLSCs + DMSO and P-PDLSCs + Osthole (10^−7^ Mol/L). Simultaneously, no defects and defects without anything served as the control groups. After implantation, penicillin was administered by muscular injection at 25,000 U/kg body weight. Rats were euthanized by excess anesthetic 4 weeks later and the mandible samples were harvested and fixed in 4% paraformaldehyde solution for 24 h. Then the samples were defined by microCT.

### MicroCT

Samples from the inferior alveolar defect mode were scanned using a Siemens Inveon Microcomputed Tomography (Micro-CT) System (Siemens, Munich, Germany) with the following scanning parameters: 30 μm resolution, 80 kV and 500 μA. The obtained data were transferred to a workstation and then three dimensional (3D) images were reconstructed by the system software. Mean area of defects was assessed by built-in software. Statistical images were presented by Prism 5.0.

### Immunohistochemistry (IHC)

To quantify the expression of MOZ and MORF in cell sheet, specimens were processed using identical protocols. Briefly, paraffin embedded tissue sections were de-waxed in xylene and rehydrated through graded alcohols to water. Endogenous peroxidase was blocked using 3% H_2_O_2_ for 15 minutes. For antigen retrieval, 10% citrate buffer was used in high-pressure boiler for 15 minutes. Specimens were blocked with 10% serum for 30 minutes, then incubated with primary antibody anti-MOZ (#sc-5713), anti-MORF (#sc-368690) (Santa Cruz Biotechnology, CA, USA) for two hours. Secondary antibody was applied for one hour at room temperature. Specimens were then incubated in strept avidin-biotin complex (SABC) (Boster, Wuhan, China) for 30 min. Diaminobenzidine (DAB) solution was applied for 2 min and development of the color reaction was monitored microscopically. Specimens were counterstained with hematoxylin, dehydrated, cleared and then mounted. Specimens were observed under a light microscope, and images of each sample were acquired randomly using a CDD camera. Quantification of MOZ/MORF-positive staining was carried out using the software of Image-Pro Plus 6.0, and images were presented by Prism 5.0.

### Statistical analyses

Data from triplicate *in vitro* experiments are presented as mean ± S.D. The Student’s t-test was used for comparing two groups of data at *p* < 0.05, *p* < 0.01 or *p* < 0.001 level of significance. Two-way analysis of variance (ANOVA) in conjugation with Tukey’s test was used to compare multiple groups of data at *p* < 0.05, *p* < 0.01 or *p* < 0.001 level of significance. All of the statistical testing results were determined by Prism 5. The Student’s t-test was performed on MTT, quantification of ARS, qRT-PCR in comparison of two groups, quantification of H & E, and quantification of IHC. Two-way ANOVA was performed on qRT-PCR in comparison of three groups, qRT-PCR of acetylases screening.

## Electronic supplementary material

Supplementary Information
