# Author Correction: Osthole improves function of periodontitis periodontal ligament stem cells via epigenetic modification in cell sheets engineering

**DOI:** 10.1038/s41598-022-10471-x

**Published:** 2022-05-31

**Authors:** Jin Sun, Zhiwei Dong, Yang Zhang, Xiaoning He, Dongdong Fei, Fang Jin, Lin Yuan, Bei Li, Yan Jin

**Affiliations:** 1 State Key Laboratory of Military Stomatology & National Clinical Research Center for Oral Diseases & Shaanxi Key Laboratory of Oral Diseases, Center for Tissue Engineering, Fourth Military Medical University, Xi’an, 710032 Shaanxi China; 2 Xi’an Institute of Tissue Engineering and Regenerative Medicine, Xi’an, 710032 Shaanxi China; 3 Department of Stomatology, The Affiliated Shenzhen Maternity and Child Healthcare Hospital of the South Medical University, Shenzhen, 518048 Guangdong China; 4 Department of Oral and Maxillofacial Surgery, General Hospital of Shenyang Military Area Command, Shenyang, 110840 Liaoning China; 5 Department of Orthopaedics, Xijing Hospital, the Fourth Military Medical University, Xi’an, 710032 Shaanxi China; 6 Department of Orthodontics, School of Stomatology, Fourth Military Medical University, Xi’an, 710032 Shaanxi China; 7 Department of Stomatology, the First Affiliated Hospital of Guangzhou Medical University, Guangzhou, 510140 Guangdong China

Correction to: *Scientific Reports* 10.1038/s41598-017-05762-7, published online 12 July 2017

This Article contains errors in Figure 1 and Figure 2.

**Figure 1:**
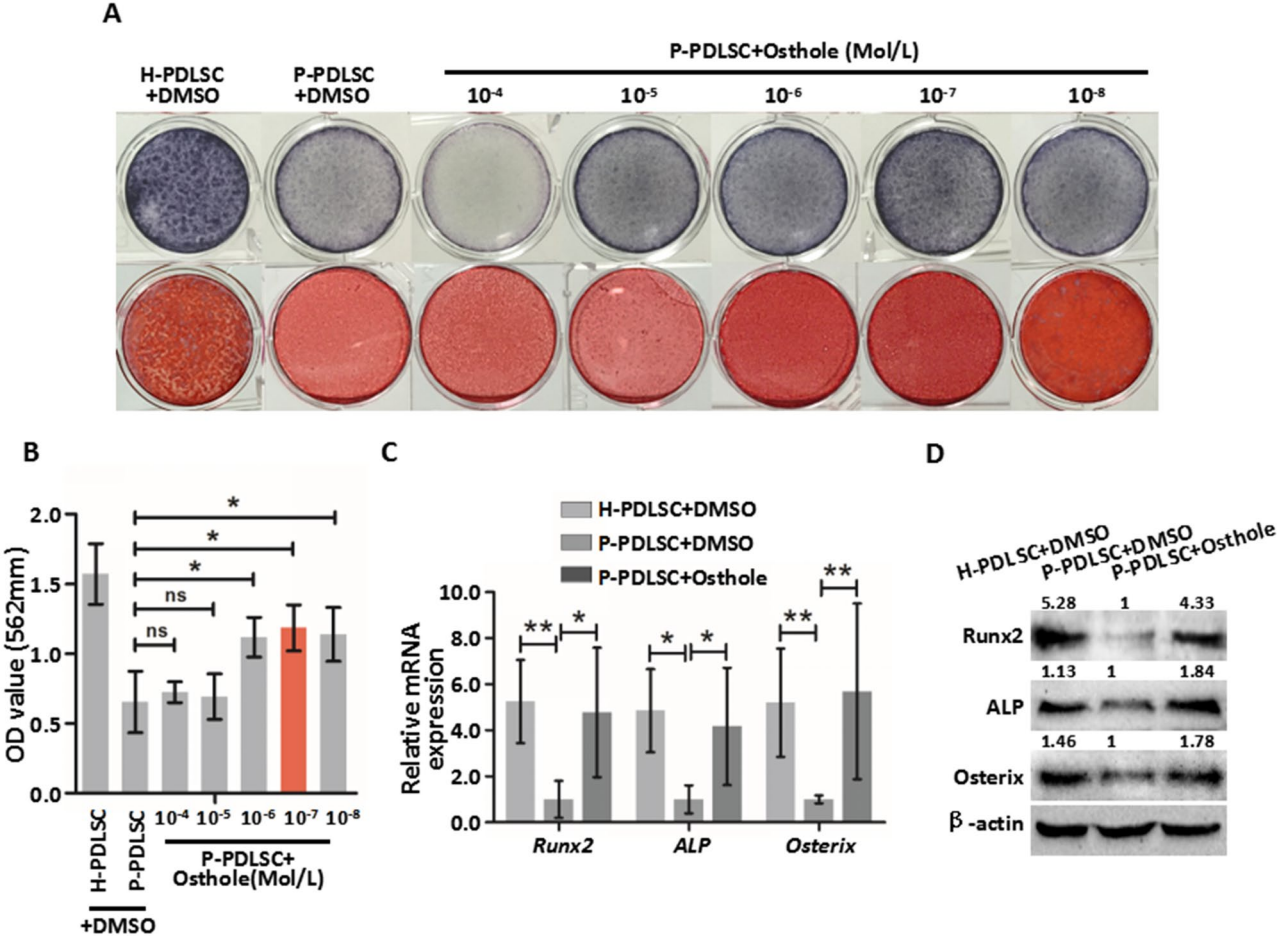
Osthole reverses defective osteogenic ability of P-PDLSCs. (**A**) ALP staining and ARS staining after 7 days (ALP staining) and 21 days (ARS staining) in H-PDLSCs and P-PDLSCs with different concentrations of Osthole (0 Mol/L, 10^−4^ Mol/L, 10^−5^ Mol/L, 10^−6^ Mol/L, 10^−7^ Mol/L and 10^−8^ Mol/L). (**B**) Quantification of ARS staining for light absorbance at 562 nm. (**C**) Gene expression of Runx2, ALP and Osterix in H-PDLSCs, P-PDLSCs and P-PDLSCs with 10^−7^ Mol/L Osthole as assayed by qRT-PCR. (**D**) Protein expression of Runx2, ALP and Osterix in H-PDLSCs, P-PDLSCs and P-PDLSCs with 10^−7^ Mol/L Osthole as assayed by Western blot. *P < 0.05, **P < 0.01, ns: *P* ≧ 0.05, n = 3.

**Figure 2:**
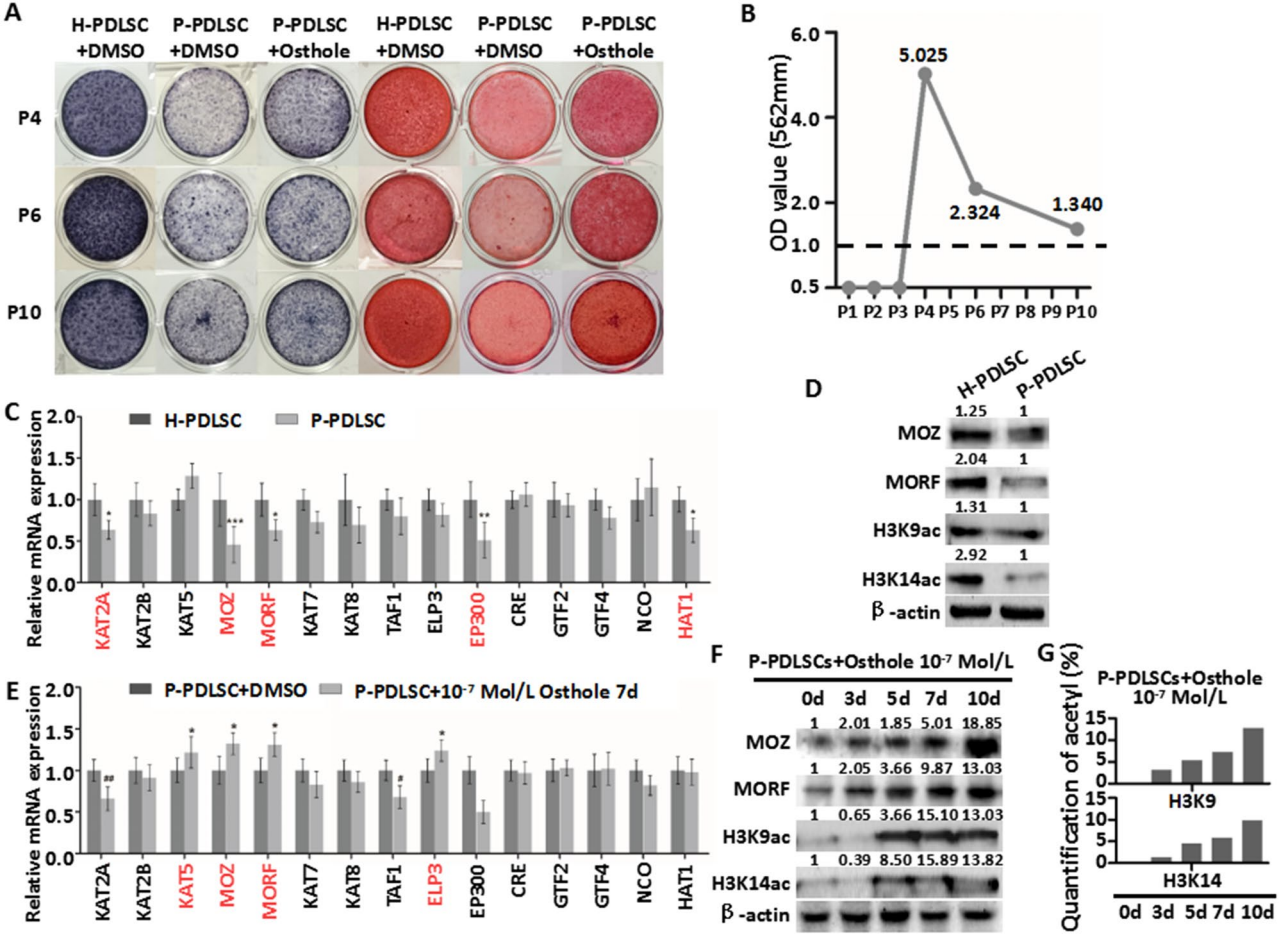
Osthole reverses defective osteogenesis of P-PDLSCs through histone acetylation. (**A**) ALP staining and ARS staining of H-PDLSCs, P-PDLSCs and P-PDLSCs with 10^−7^ Mol/L Ostholes in P4 (with stimulation), P6 (without stimulation) and P10 (without stimulation). (**B**) Quantification of ARS staining for light absorbance at 562 nm. (**C**) qRT-PCR showed gene expression of fifteen histone acetylases in H-PDLSCs and P-PDLSCs. (**D**) Protein expression of MOZ, MORF, H3K9ac and H3K14ac in H-PDLSCs and P-PDLSCs as assayed by western blot. (**E**) qRT-PCR showed gene expression of fifteen histone acetylases in P-PDLSCs and P-PDLSCs with 10^−7^ Mol/L Osthole measured by qRT-PCR. (**F**) Protein expression of MOZ, MORF, H3K9ac and H3K14ac in P-PDLSCs with 10^−7^ Mol/L Osthole treatment on day 0, 3, 5, 7, 10 as assayed by western blot. (**G**) Level of acetylation of H3K9 and H3K14 in P-PDLSCs with 10^−7^ Mol/L Osthole treatment on day 0, 3, 5, 7, 10 as assayed by EpiQuik Global Acetyl Histone Quantification Kit. *P < 0.05, **P < 0.01, ***P < 0.001, no mark: *P* ≧ 0.05, n = 3.

In Figure 1A, the ARS staining images in the lower row for H-PDLSC+DMSO and P-PDLSC+Osthole, 10^−8^ Mol/L are incorrect. Furthermore, in Figure 2A, the APS straining image for H-PDLSC+DMSO in P6 is incorrect. The correct Figure 1 and Figure 2 and accompanying legends appear below.

