# Supplementary material for: Osthole improves function of periodontitis periodontal ligament stem cells via epigenetic modification in cell sheets engineering
Source: Sci Rep. 2017 Jul 12;7:5254. doi: 10.1038/s41598-017-05762-7 (PMC5507976; doi:10.1038/s41598-017-05762-7)
Supplement: Supplementary file 1 — Supplementary Information [file 41598_2017_5762_MOESM1_ESM.pdf]

# Osthole improves function of periodontitis periodontal ligament stem cells via epigenetic modification in cell sheets engineering

Jin Sun, Zhiwei Dong, Yang Zhang, Xiaoning He, Dongdong Fei, Fang Jin, Lin Yuan, Bei Li, Yan Jin

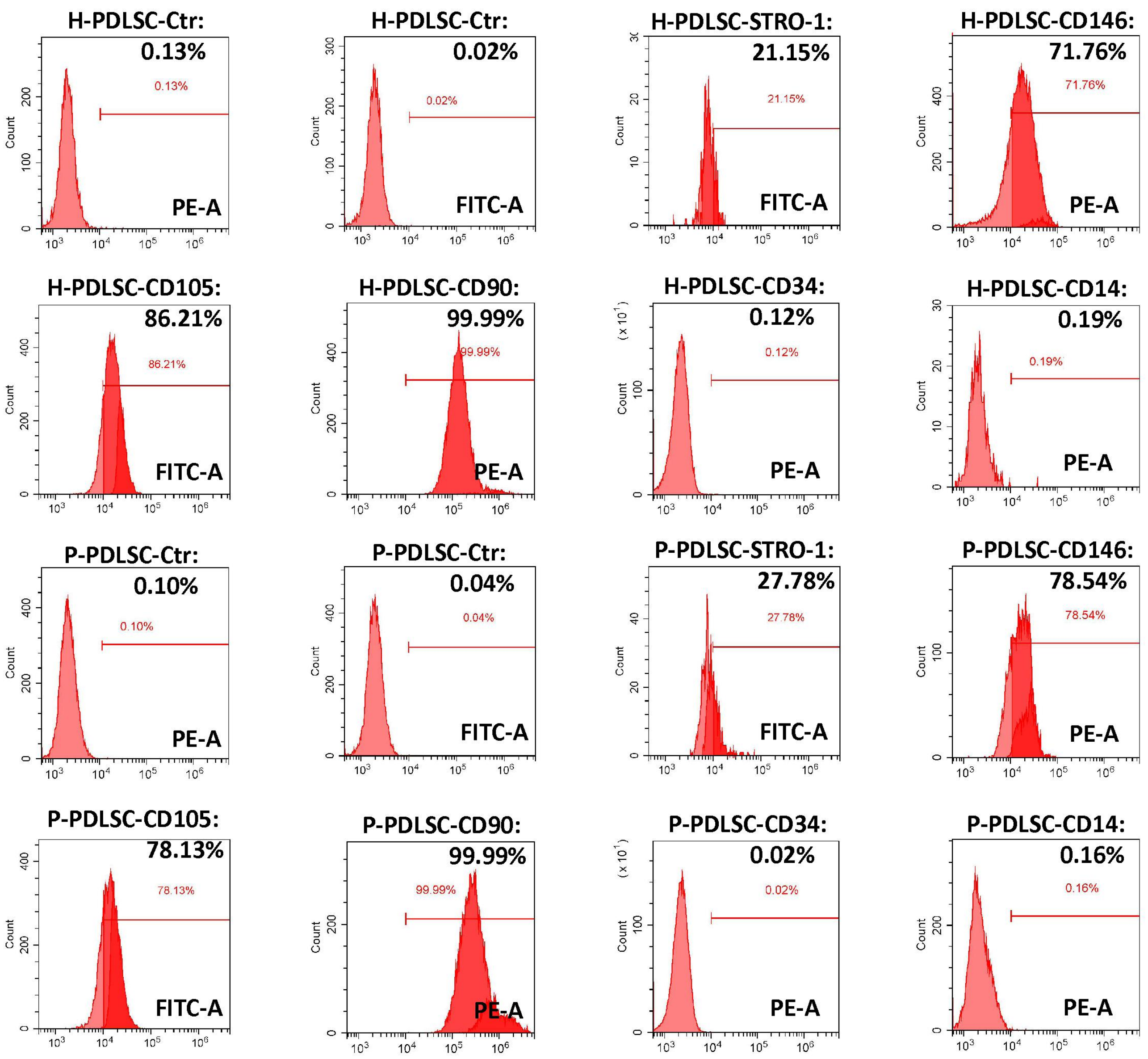


Supplementary figure 1

Flow cytometry analysis of MSC-markers. Analyses were performed via flow cytometry detecting PE or FITC conjugated monoclonal antibodies for human Stro-1, CD146, CD105, CD90, CD34, CD 14 or isotype matched control IgGs. Ctr: control.

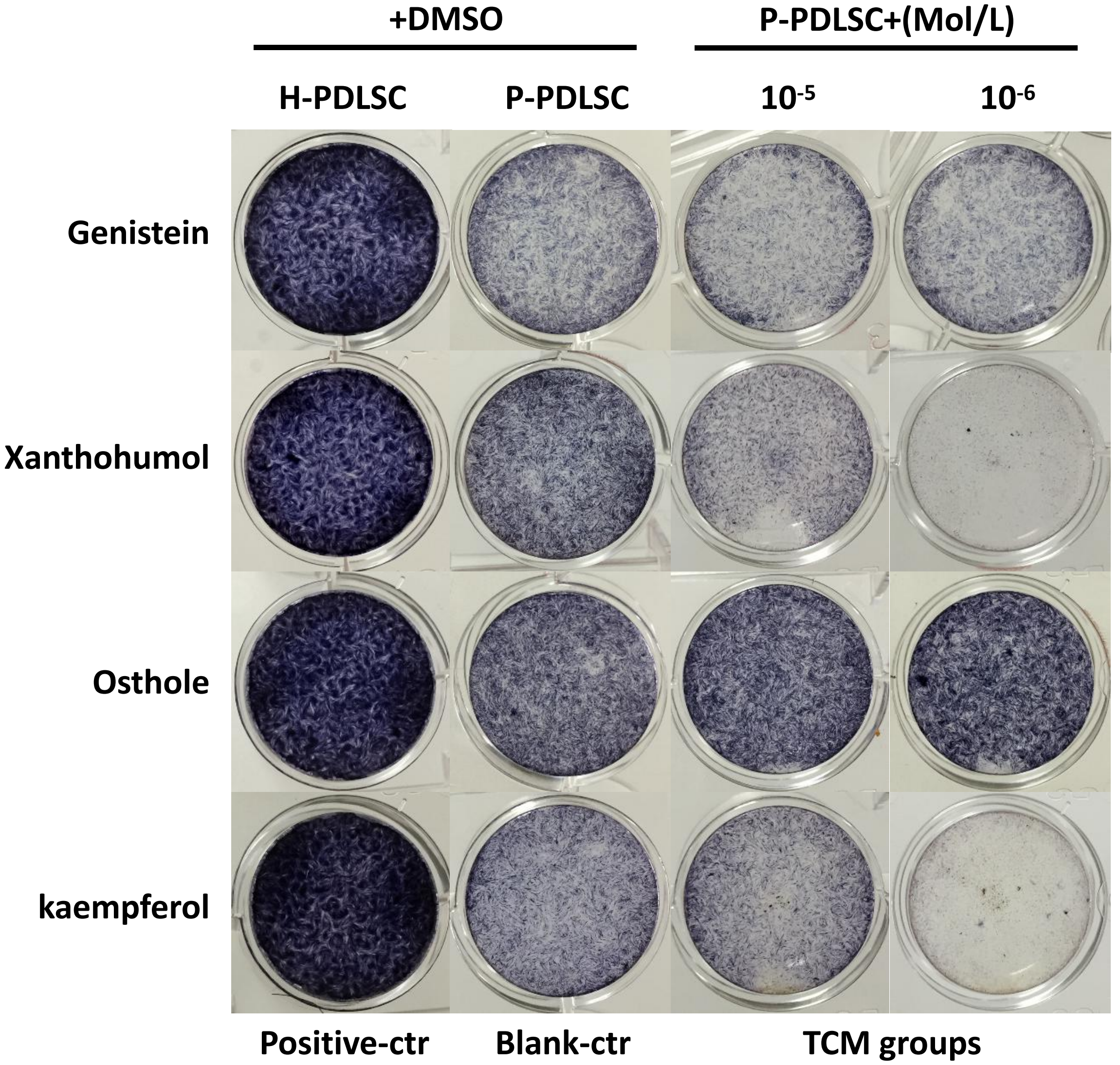


Supplementary figure 2
Osteogenic effect of TCMs on P-PDLSCs. First panel showed cells with Genistein treatment. Second panel showed cells with Xanthohumol treatment. Third panel showed cells with Osthole treatment. Fourth panel showed cells with Kaempferol treatment. Positive-ctr: positive control group, Blank-ctr: blank control.

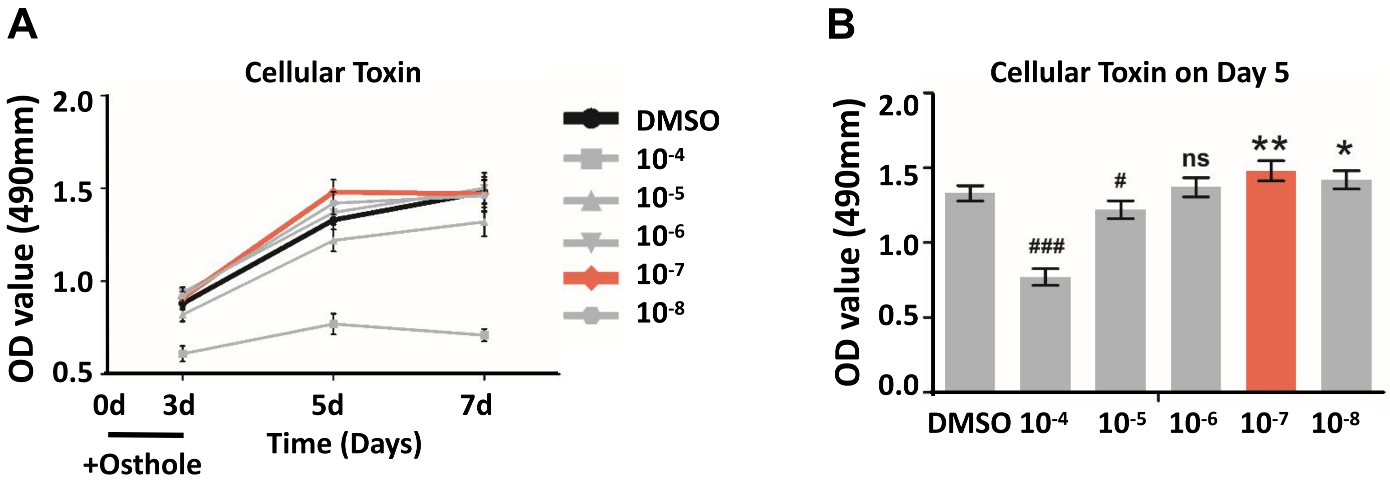


Supplementary figure 3
Proliferation of P-PDLSCs after Osthole treatment. (A) MTT assay of P-PDLSCs with different concentrations of Osthole (0 Mol/L, 10-4 Mol/L, 10-5 Mol/L, 10-6 Mol/L, 10-7 Mol/L and 10-8 Mol/L) on day 3, 5 & 7. (B) MTT assay of P-PDLSCs with Osthole treatment of the same concentrations as above on day 5. */#: $P < 0.05$, **: $P < 0.01$, ###: $P < 0.001$, ns: $P \geqq 0.05$, $n = 3$.

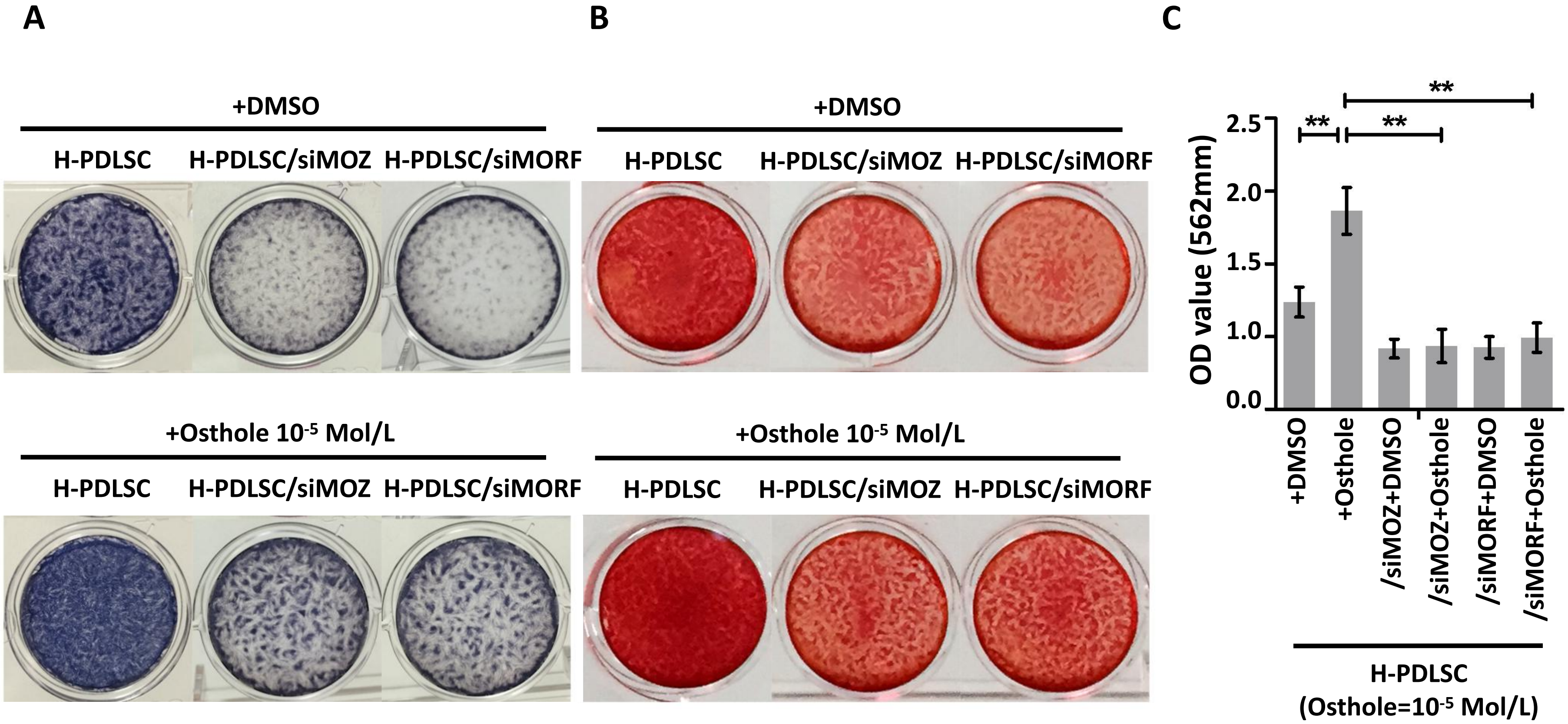


Supplementary figure 4
Osteogenic differentiation of H-PDLSCs/siMOZ or H-PDLSCs/siMORF with $10^{-5}$ Mol/L Osthole. (A) ALP staining in H-PDLSCs with Osthole treatment after 7 days osteogenic induction. (B) ARS staining in H-PDLSCs with Osthole treatment after 21 days osteogenic induction. (C) Quantification for light absorbance at 562 nm. **: $P < 0.01$, $n = 3$.
